# SMPD1基因两个位点错义突变的尼曼匹克病1例报告并文献复习

**DOI:** 10.3760/cma.j.cn121090-20251108-00513

**Published:** 2026-03

**Authors:** 岩 尉, 红 郭, 嘉宁 徐, 育红 陈, 红霞 石

**Affiliations:** 1 北京大学人民医院，北京大学血液病研究所，国家血液系统疾病临床医学研究中心，造血干细胞移植治疗血液病北京市重点实验室，北京 100044 Peking University People's Hospital, Peking University Institute of Hematology, National Clinical Research Center for Hematologic Disease, Beijing Key Laboratory of Hematopoietic Stem Cell Transplantation, Beijing 100044, China; 2 青岛市妇女儿童医院检验科，青岛 266113 Clinical Laboratory, Women and Children's Hospital, Qingdao 266113, China

## Abstract

本文报道1例SMPD1基因双位点错义突变所致尼曼匹克病并探讨尼曼匹克细胞与海蓝组织细胞的形态学关联。患者骨髓涂片可见典型尼曼匹克细胞、含少量至中等量海蓝颗粒的尼曼匹克细胞及海蓝组织细胞。二代测序基因分析显示患者SMPD1杂合突变c.1361C>A（p.Ala454Asp）、c.1666C>T（p.His556Tyr），该类突变既往仅见文献报道，临床意义未明。SMPD1基因双位点错义突变具有致病性，可导致尼曼匹克病。推测尼曼匹克细胞与海蓝组织细胞存在形态学关联，联合骨髓形态学与基因检测有助于提升该病及相关脂质贮积病的精准诊断水平。

## Abstract (en)

This report describes a case of Niemann-Pick disease caused by double missense mutations in the SMPD1 gene and investigates the morphological relationship between Niemann-Pick cells and sea-blue histiocytes. The patient's bone marrow smear showed typical Niemann-Pick cells, containing small to moderate sea-blue granules, as well as sea-blue histiocytes. Next-generation sequencing revealed heterozygous SMPD1 mutations c.1361C>A（p. Ala454Asp）and c.1666C>T（p. His556Tyr）. While these mutations were previously reported in the literature, their clinical significance remains unclear. Since missense mutations in SMPD1 are pathogenic and can cause Niemann-Pick disease, it is hypothesized that a morphological link exists between Niemann-Pick cells and sea-blue histiocytes. Combining bone marrow morphology with genetic testing can improve the diagnostic accuracy for this disease and other related lipid storage disorders.

尼曼匹克病（NPD）是一组较为罕见的常染色体隐性遗传、多系统受累的疾病，主要由于编码神经鞘磷脂酶的基因突变，致使神经鞘磷脂酶缺乏，神经鞘磷脂无法水解，进而沉积于单核-巨噬细胞系统和神经组织细胞中，形成特征性尼曼匹克细胞。临床上，NPD主要分为NPD-A/B型和NPD-C型。其中，NPD-A/B型是由鞘磷脂磷酸二酯酶1（SMPD1）基因突变导致酸性鞘磷脂酶（ASM）缺乏所引起；NPD-C型则是由NPC细胞内胆固醇转运蛋白1（NPC1）或NPC细胞内胆固醇转运蛋白2（NPC2）基因突变造成胆固醇转运和酯化障碍所致[1]–[2]。NPD-A型为婴儿型，是NPD最为严重的亚型，其首发表现为器官肿大，常伴有严重的神经系统损害，生长发育迟缓，大多数患儿可检测到黄斑区樱桃红色斑点，患者多在婴幼儿期死亡[3]。NPD-B型为慢性非神经型，病情进展呈现异质性，主要表现为肝脾肿大、肺部病变、高脂血症等。血小板计数减少是其典型表现[4]。NPD-C型中约95％的病例是由NPC1基因突变引起，5％是由NPC2基因突变引起。NPD-C型的临床表现呈现多样性，包括神经系统和全身系统症状[5]。本文报道1例NPD患者诊疗过程以及SMPD1基因检测结果并进行相关文献复习。

## 病例资料

患者，女性，65岁。因“发现脾大伴间断咳嗽、活动后气短1年余”就诊于北京大学人民医院。患者自述30年前检查发现脾大，但无其他症状及临床表现，未予治疗，仅规律随访。2024年2月因新型冠状病毒感染查腹部超声提示脾大（长径22.7 cm，厚径6.5 cm）。PET-CT提示巨脾伴代谢弥漫轻度增高，双肺胸膜下多发代谢轻度增高斑片网格影，双肺多发局限性肺气肿、肺大疱，双肺尖胸膜增厚，左侧伴钙化。予抗病毒治疗。近1年来患者逐渐出现间断咳嗽，咳少量白痰，活动后气短，休息后可缓解，无发热、胸痛、咯血等症状。无神经系统异常。2024年6月为行切脾就诊于北京大学人民医院，复查腹部彩超显示：脾厚约6.2 cm，长径约24 cm。既往史：曾患黑热病，接受锑剂治疗而愈；间质性肺炎17年，目前口服泼尼松治疗。有结核病及风湿热病史。家族史：同胞姐姐因黑热病病逝。CT增强扫描显示肝大、巨脾及肺间质改变。血常规：WBC 5.7×10^9^/L、HGB 112 g/L、PLT 90×10^9^/L。骨髓细胞学：骨髓增生活跃，可见大量海蓝组织细胞，胞体偏大、核居中或偏位、核染色质聚集、胞质丰富，含较多海蓝色颗粒。骨髓组织学：骨小梁间区组织细胞片状分布，核规则、居中或偏位、偶见双核，胞质丰富、颗粒状；造血组织红系、粒系细胞呈灶性增生；前体细胞可见，两系中、晚阶段细胞散在或可见小堆；巨核细胞0～2个；淋巴细胞、浆细胞、嗜酸细胞可见（图1）。Gomori：MF-1。ASM 1.8 nmol·g^−1^·min^−1^（正常参考范围：7.0～20.8 nmol·g^−1^·min^−1^），β-半乳糖苷酶282.9 nmol·g^−1^·min^−1^（正常参考范围：144.7～350.4 nmol·g^−1^·min^−1^），ASM酶活力检测结果偏低，提示NPD-A或NPD-B型。二代测序外显子基因分析患者SMPD1杂合突变c.1361C>A（p.Ala454Asp）、c.1666C>T（p.His556Tyr），c.1361C>A（p.Ala454Asp）为SMPD1基因编码区错义变异，该基因相关联疾病为NPD，结合患者年龄及临床症状，考虑NPD-B型可能性大。患者因间质性肺炎导致肺功能受损，耐受度不佳，暂未实施脾切除术。针对NPD，目前亦未行特异性治疗。患者坚持规律随访至2025年12月，随访期间病情整体平稳，存在肺功能减退、脾大伴腹胀不适及关节软组织疼痛等症状，未见明显疾病进展及严重不良事件发生。

**图1:**
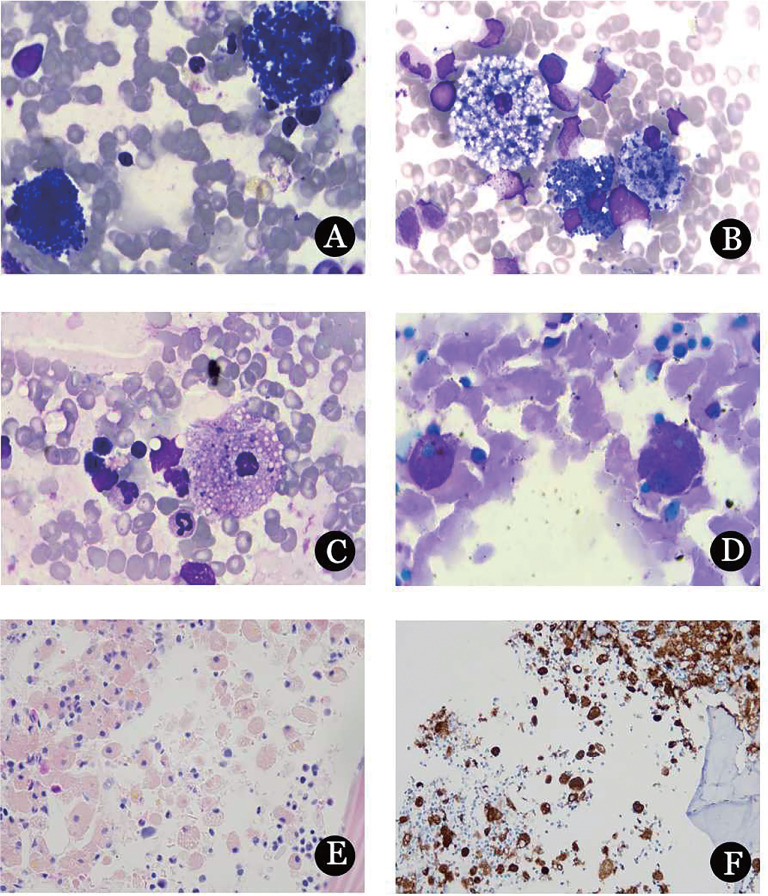
尼曼匹克病患者骨髓涂片形态及骨髓活检染色结果 **A** 典型的海蓝组织细胞（Wright-Giemsa染色，×1 000）；**B** 尼曼匹克细胞（含有海蓝色颗粒）与海蓝组织细胞（Wright-Giemsa染色，×1 000）；**C** 典型尼曼匹克细胞（Wright-Giemsa染色，×1 000）；**D** PAS染色阳性（×1 000）；**E** HE染色（×400），组织细胞片状分布，核规则、居中或偏位、偶见双核，胞质丰富、颗粒状；**F** CD68免疫组织化学染色（×400），阳性率为60％

## 讨论及文献复习

本例患者为老年起病，临床主要表现为脾大合并肺间质病变，病程中无显著神经系统受累症状及体征。骨髓细胞形态学检查可见海蓝组织细胞（初诊拟诊颗粒型海蓝组织细胞）、含少量至中等量脂质的尼曼匹克细胞（初诊拟诊颗粒泡沫型海蓝组织细胞）及典型尼曼匹克细胞（初诊拟诊泡沫型海蓝组织细胞），初步提示海蓝组织细胞增多症（SBH）。为明确病因，进一步行ASM及β-半乳糖苷酶活性检测，结果提示ASM活性降低。全外显子基因检测检出患者携带SMPD1杂合突变，分别为c.1361C>A（p.Ala454Asp）及c.1666C>T（p.His556Tyr）。上述2个突变位点均未收录于大规模人群数据库，临床意义暂未明确。致病性生物信息学分析结果如下：SMPD1 c.1361C>A（p.Ala454Asp）即SMPD1 c.1361C>A（p.A454D）位点经ClinVar数据库注释为可能致病性变异，经23种生物信息学软件功能预测，22种软件判定该突变为有害突变，有害预测比例达95.65％。蛋白结构层面分析提示，该位点野生型氨基酸为丙氨酸，属疏水性小分子氨基酸，突变后变为天冬氨酸，为带负电荷的大分子氨基酸；若该位点位于蛋白质内部疏水核心区，突变引入的电荷效应可显著破坏蛋白质空间结构稳定性，提示该变异具有明确的蛋白功能破坏潜能，文献报道在NPD患者中检测到该变异[6]。SMPD1 c.1666C>T（p.His556Tyr）即SMPD1 c.1666C>T（p.H556Y）位点经ClinVar数据库注释为临床意义未明变异；经23种生物信息学软件功能预测，14种软件判定该突变为有害突变，有害预测比例为60.87％。蛋白结构层面分析提示，该位点野生型氨基酸为组氨酸，属带正电的极性氨基酸，突变后变为酪氨酸，为含苯环结构的大分子氨基酸；若突变位点所处空间构象狭窄，可能产生空间位阻效应，整体预测结果偏良性，但仍不能完全排除其对蛋白质功能的潜在影响。综合形态学、酶学检测、基因测序及生物信息学分析结果，SMPD1 p.A454D变异极可能导致编码蛋白结构与功能异常，p.H556Y变异虽整体预测偏良性，仍存在潜在的蛋白功能损伤效应。本例患者的临床表型与分子检测结果相互印证，为SMPD1基因变异的致病性判定提供了新的临床病例依据。

临床实践中，NPD易与以下两类组织细胞相关疾病混淆[7]–[8]：戈谢病，为β-葡萄糖苷酶（GBA）基因缺陷所致的常染色体隐性遗传性溶酶体贮积病[9]，致病突变导致GBA活性降低，葡萄糖脑苷脂无法正常降解而异常沉积于单核-巨噬细胞系统，形成特征性戈谢细胞。戈谢细胞体积巨大，胞质丰富，呈淡粉色至灰蓝色，胞质内可见特征性淡蓝紫色条纹状结构，呈洋葱皮样或蛛网状排列；胞核较小，可为单个或多个，核染色质粗糙致密、浓集深染，偶见核仁结构。SBH以成熟分化的组织细胞良性增生为主要特征，巨噬细胞胞质内充满特征性蓝绿色不透明颗粒，因颗粒色泽近似海水蓝色而得名。海蓝组织细胞体积较大，呈圆形或椭圆形，胞质丰富，内含数量不等、大小均一的海蓝色折光颗粒，胞核较小，多为单核，偶见双核或三核，核常偏位，染色质呈粗网状分布。依据形态学特征，海蓝组织细胞可分为四型：颗粒型、颗粒泡沫型（混合型）、泡沫型及退化型（网架型）[10]。本例患者细胞的形态学表现与文献报道的海蓝组织细胞类型存在形态重叠，是初始形态学诊断提示海蓝组织细胞增多的核心依据。鉴于原发性SBH临床极其罕见，且其临床表现、病理特征与NPD存在高度重叠，病因及发病机制尚未完全阐明，据此推测本例患者骨髓中出现的海蓝组织细胞本质上可能为尼曼匹克细胞的特殊形态学亚型。

当前学界对二者关系存在差异观点：国内多项研究[11]–[12]将该类表型定义为NPD伴继发性海蓝组织细胞增多，而国外学者则提出，海蓝组织细胞本质即为尼曼匹克细胞的特殊形态变异型[13]。细胞化学染色证实，两类细胞胞质内均蓄积大量糖类与脂质成分。就其超微结构而言，海蓝组织细胞显示为类脂分子呈周状板层结构或不同的脂质沉积，尼曼匹克细胞显示膜结合的同心板层小体（类似髓磷脂样小体）或平行板层形状。两类细胞超微结构的高度相似性，强烈提示其胞内贮积物均具备磷脂样物理化学特性。基于上述证据，本研究提出推测：因溶酶体水解酶缺陷导致机体糖脂代谢通路受阻，神经鞘磷脂、神经鞘糖脂等脂质成分过量贮积于单核-巨噬细胞系统，而吞噬细胞内贮积脂质的种类、比例及含量差异，最终决定细胞呈现不同形态表型，即海蓝组织细胞以神经鞘糖脂贮积为主、神经鞘磷脂为辅，故PAS染色呈阳性；而尼曼匹克细胞以神经鞘磷脂贮积为主、神经鞘糖脂为辅，故PAS染色仅颗粒边缘呈阳性反应。该假说可合理解释临床中NPD常合并海蓝组织细胞增多、既往诊断为SBH的病例亦可检出尼曼匹克细胞的双向表型重叠现象。本文仅为个案报道，存在局限性，海蓝组织细胞的病理本质、其与尼曼匹克细胞的谱系同源性及形态转化机制，未来仍需更大样本的临床研究、分子病理学及超微结构分析予以进一步阐明与验证。
